# Sonographic Assessment of Fetometric Parameters in Pigs of Different Prolific Genotypes for Gestational Age Estimation

**DOI:** 10.3390/ani16020349

**Published:** 2026-01-22

**Authors:** Frauke Janelt, Johannes Kauffold, Haukur Lindberg Sigmarsson, Ahmad Hamedy, Katharina Riehn, Martin Koethe, Jörg Altemeier, Philipp Maximilian Rolzhäuser

**Affiliations:** 1 Department Meat Hygiene, Institute of Food Hygiene, Faculty of Veterinary Medicine, Leipzig University, 04109 Leipzig, Germany; 2 Clinic for Ruminants and Swine, Faculty of Veterinary Medicine, Leipzig University, 04109 Leipzig, Germany; 3 Faculty of Life Sciences, Hamburg University of Applied Sciences, 21033 Hamburg, Germany; 4 Animal Welfare Department, Premium Food Group ApS & Co. KG, 33378 Rheda-Wiedenbrück, Germany

**Keywords:** fetus, pregnancy, pig, ultrasonography

## Abstract

The slaughter of pregnant sows remains a relevant concern in modern swine production, with prevalence rates reported from 1.5–13% in Europe. Considering fetal sensitivity during late gestation and legal restrictions on transport and slaughter, reliable assessment of fetal age is of considerable practical, ethical, and legal relevance. In this study, 70 pregnancies from low-prolificacy (purebred German Saddleback) and medium-to-high prolificacy genotypes (purebred German Landrace and Duroc × German Landrace hybrids) were repeatedly examined using transabdominal ultrasonography, with a total of 15 examinations per pregnancy. Seven fetometric parameters—rosto-occipital distance, bi-parietal distance, orbital distance, sternum length, thorax diameter, body diameter, and crown–rump length—were measured in vivo, assessing two fetuses per pregnancy and calculating mean values to account for intra-individual variation. Parameter feasibility varied across gestation: during early gestation (gestation days 38 and 40), orbital distance, sternum length, and crown–rump length could be reliably measured; in mid-gestation, all seven parameters were measurable, whereas in late gestation (from gestational day 87 onward), crown–rump length was no longer measurable, and the remaining six parameters remained assessable for gestational age estimation. Crown–rump length (CRL) increased from a median of 3.2 cm (range 1.9–4.2 cm) at day 38 to 16.3 cm (range 14.0–18.2 cm) at day 77, representing the most practical parameter for determining the stage of gestation. Litter size had no significant effect on fetometric growth, except for a weak correlation with thorax diameter at day 77, and parity showed no measurable influence on any parameter. The results show that fetometric values in modern sow genotypes are smaller than those reported in earlier literature, highlighting the need for updated gestational age assessment. These findings provide practical guidance for gestational age estimation, supporting the enforcement of animal welfare legislation and potentially contributing to a reduction in the slaughter of highly pregnant sows.

## Simple Summary

The slaughter of pregnant pigs remains a major animal welfare concern, as animals may be transported or slaughtered at advanced stages of pregnancy when fetuses are particularly sensitive to pain or discomfort. To prevent this, it is essential to reliably determine how far pregnancy has progressed. However, much of the available information is based on older studies and does not reflect modern pig production. The aim of this study was to provide updated measurements of fetal growth in pigs under current farming conditions. Using ultrasound examinations during pregnancy, fetal size was measured repeatedly in a large number of animals. The results showed that fetal growth follows consistent patterns and that specific measurements can be used to estimate the stage of pregnancy at different times. These findings provide practical guidance for veterinarians and authorities when assessing pregnancy stage in pigs and support better decision-making to protect animal welfare.

## 1. Introduction

The slaughter of pregnant pigs remains a significant concern in modern livestock production [1,2,3,4], as it raises considerable animal welfare issues. Although the ability of late-term pig fetuses to perceive pain, distress, or discomfort has not been conclusively demonstrated, it cannot be excluded [1]. In light of this uncertainty, the precautionary principle warrants careful scrutiny of slaughter practices involving animals in mid to late gestation [5]. Beyond scientific debate, public perception strongly opposes the slaughter of pregnant livestock, emphasizing the ethical relevance of this practice [6]. Regulation (EC) No. 1/2005 prohibits the transport of pregnant animals that have reached 90% or more of the expected gestation period [7]. In Germany, the Animal Products Trade Prohibition Act restricts the delivery of mammals in the final third of gestation for slaughter [8]. Although the slaughter of pregnant pigs is not explicitly forbidden, these provisions demand accurate determination of gestational age for ensuring compliance and guiding veterinary decisions. Available prevalence data is largely based on older studies in which pregnancy detection was incidental rather than the primary objective. European studies report prevalence rates ranging from 1.5% to 13% in pigs presented for slaughter [2,4,9,10,11]. The European Food Safety Authority (EFSA) estimated an European Union (EU)-wide prevalence of 6%, including 0.5% of pigs slaughtered during the final trimester of gestation [1]. In Germany, a single study documented an overall prevalence of 3%, with approximately 0.3% of sows and gilts affected in the final trimester [3]. Scientific literature on fetal development in pigs remains sparse and largely outdated. In practice, gestational age in sows is commonly estimated based on breeding or insemination records. Post-mortem determination has traditionally relied on morphometric parameters such as crown–rump length [12,13,14,15,16], fetal weight [17,18,19], and the assessment of organ development [20] or skeletal mineralization [21]. In Germany, fetal age determination in pigs has so far been conducted exclusively on deceased fetuses, with most reference data relying on the compilation by Schnorr and Kressin [19], which draws on data from Habermehl (1980) [15] and Evans & Sack (1973) [16]. In these studies, crown–rump length was measured with the body in a physiological, slightly flexed position. This approach uses rounded gestational thresholds rather than mathematically derived trimester limits and introduces variability that limits reproducibility. In addition, information on the breed and the assumed gestational length is lacking. Fetometric data and ultrasonographic studies for fetal age estimation have also been reported in other species, including goats [22,23,24,25,26,27,28,29], sheep [30,31,32], cattle [33,34,35], and horses [36,37]. However, no studies to date have investigated the use of ultrasonography for in vivo age estimation of pig fetuses. Moreover, with genetic progress increasing litter size and altering fetal growth dynamics, the relevance of historical data for modern commercial sow herds is increasingly questionable, as smaller fetuses within larger litters may develop at a lower rate, complicating direct comparisons. The present study aimed to use ultrasonography to generate fetometric parameters for age determination in swine, comparing different prolific genotypes, and to establish criteria applicable to modern hybrid lines.

## 2. Materials and Methods

### 2.1. Farm, Animals and Animal Management

The study was conducted on the Research and Teaching Farm of the Leipzig University between January 2024 and February 2025. The farm operated as a farrow-to-finish unit with an inventory of 40 reproductive females. In this study, a total of 70 pregnancies were monitored (sows: *n* = 57; parity 1–9, distribution: 1st parity: 14, 2nd: 15, 3rd: 11, 4th: 8, 5th: 4, 6th: 2, 7th–9th: 1 each; gilts: *n* = 13), with the third of females being examined in two consecutive pregnancies (*n* = 26). The sows and gilts represented the following genotypes: purebred German Landrace (GL) (*n* = 38) and Duroc × German Landrace hybrids (DuGL) (*n* = 12), classified as medium- to high-prolificacy genotypes based on breeding performance, and purebred German Saddleback (GS) (*n* = 20), a low prolificacy genotype resembling older genetic lines with characteristically small litter sizes. The lower number of German Saddleback sows and gilts compared with German Landrace and Duroc × German Landrace hybrids reflected their reduced overall proportion within the herd, accounting for approximately 20% of each insemination group.

Breeding was performed according to on-farm Standard Operating Procedures (SOPs). Sows were weaned on Thursday AM and received daily boar contact starting Saturday until the end of breeding. Sows found in estrus were immediately bred by artificial insemination (AI) using commercial semen of different breeds (Piétrain, German Landrace, German Saddleback, and Duroc) and again at midday of the same day. If they were still in heat the next day, they received a third AI (24 h after first AI). For this study, the first insemination was considered day 0 of pregnancy. Thus, by this approach, the maximum deviation in gestational age was approximately one day, depending on ovulation. Gilts were synchronized for estrus using Altrenogest (20 mg/animal, Altresyn^®^, Ceva, Libourne, France) given orally once daily for 18 consecutive days. Breeding was performed as reported for sows by three AI in maximum.

Sows and gilts were moved into the gestation unit one day post last artificial insemination, where they were housed in groups of seven to ten per pen with adequate space allowance on partially slatted floors and, in addition, access to an outdoor area with straw as bedding and playing material, until day 107 of gestation. Up until day 107 of gestation, animals received a commercial gestation diet (11.8 MJ ME/kg; 13.5% crude protein; 0.65% lysine; 3–4 kg/day depending on body weight), with water available ad libitum. From day 108 onward, sows were relocated to the farrowing unit, which was equipped with 2 × 6 farrowing crates on fully slatted floors, and were subsequently fed a commercial lactation diet (13.0 MJ ME/kg; 16.5% crude protein; 0.95% lysine).

### 2.2. Fetometry by Ultrasonography

Fetometric examinations were performed using a ZONARE Z.One scan engine (ZONARE Medical Systems, Inc., Mountain View, CA, USA) equipped with a curved array transducer (ZONARE C9-3) operating at 6 MHz. The penetration depth was adjusted according to gestational age, ranging from 8 cm at day 38 to 18 cm at day 110 of gestation (day 38–40: 8 cm; day 47: 10 cm; day 54: 14 cm; day 58: 16 cm; from day 58 onward: 18 cm). Sonographic assessments were conducted transabdominally (transcutaneously) on crated sows, as described by Kauffold et al. [38]. Fetal growth measurements were carried out on 15 predetermined gestational days: 38, 40, 47, 54, 58, 65, 72, 74, 77, 80, 87, 94, 101, 103, and 110, with days 38 and 77 corresponding to the end of first and second trimester, respectively (based on a 114 days gestation length [39]). To account for potential variations in fetal size, two fetuses per sow or gilt were selected for measurements on each examination day. Fetuses were chosen in a lightly flexed spinal position, avoiding marked compression or overextension, to maintain the physiological alignment of anatomical structures and reduce potential measurement artifacts. Selection was further restricted to fetuses in which all anatomical structures required for the respective fetometric measurements were clearly visible. By measuring two fetuses per animal and calculating the mean value, intra-litter dependence is accounted for and the impact of potential outliers minimized, yielding a robust value for gestational age assessment. Fetuses were neither individually marked nor recognizable, so it was not possible to track or re-identify individual fetuses across subsequent examinations (from gestation day 38 to 110). Consequently, it must be assumed that different fetuses were examined at each measurement session.

The parameters chosen for this study were selected for their suitability for reliable ultrasonographic evaluation and to allow potential application under practical conditions.

**Figure 1:**
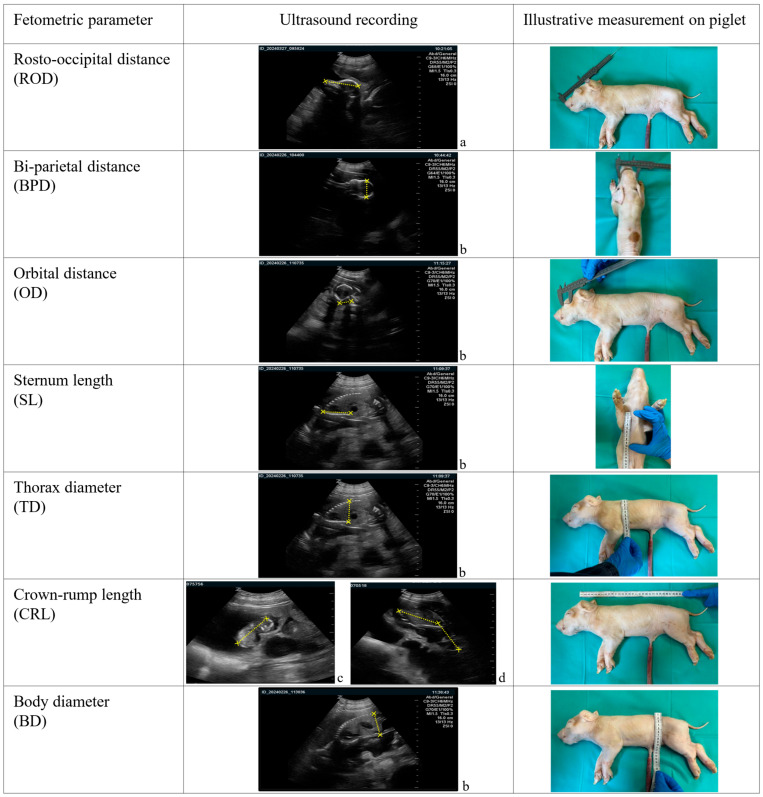
Sonographic and illustrative ex vivo measurement of fetometric parameters assessed in pig fetuses. Age of the fetus: a. 65 days, b. 72 days, c. 40 days, d. 54 days.

Per fetus, seven parameters were recorded (Figure 1):

- Skull measurements:
  ◦ Rosto-occipital distance (ROD): distance from the nasal tip to the occipital bone;
  ◦ Bi-parietal distance (BPD): widest transverse measurement between the parietal bones;
  ◦ Orbital distance (OD): diameter of the orbit.

- Thoracic measurements:
  ◦ Sternum length (SL): length of the sternum;
  ◦ Thorax diameter (TD): widest dorsoventral distance between the spine and sternum.

- Body measurements:
  ◦ Body diameter (BD): widest dorsoventral distance between the spine and the abdominal wall at the umbilical region;
  ◦ Crown–rump length (CRL): distance from the highest point of the fetal crown to the base of the rump in physiological position.

The parameter definitions were adapted from published studies in other species, specifically: ROD and BPD from sheep [31]. OD, TD, BD and CRL from goats [27,32].

When the CRL could be captured entirely in a single sonographic image, it was directly measured from the crown to the rump. For fetuses exceeding the maximum display capacity of the ultrasound machine along the longitudinal axis, the CRL was measured in two segments, following the approach described by Khand et al. [29]: The first segment from the crown to the base of the heart, the second from the base of the heart to the rump. The total CRL was then calculated as the sum of both segments.

Measurements of the relevant parameters were recorded as short ultrasound video sequences of approximately three seconds per fetus. For each gestational day, all parameters were measured once per fetus based on the corresponding video sequence, which was then retrospectively analyzed using the device’s built-in, factory-programmed software version 4.8 (ZONARE Medical Systems, Inc., Mountain View, CA, USA; software version 4.8). All measurements and retrospective analyses were performed by the same examiner to ensure consistency.

Due to the fact of a lack of reference data in swine (with only goat and sheep [27,28,29,31,32], cattle [33,34,35] and horse [36,37] data available), an attempt was made to validate the parameters by measurements in a water bath prior to the in vivo measurements. Gravid uteri (obtained from the abattoir) were examined following the methodology described by Kähn et al. [40]. This was performed using ultrasound settings identical to those used in vivo and a fixed probe-to-organ distance of 3 cm (*n* = 5, total number of fetuses: 50). Since breeding dates were not available, the gestational age was estimated to correspond to the first trimester according to Habermehl (1980) [15] and Evans & Sack (1973) [16]. After sonographic assessment, fetuses were excised from the uteri, and all of the aforementioned parameters were recorded with a measuring tape in a subsequent, independent post-mortem examination. These measurements validated the feasibility and accuracy of subsequent in vivo ultrasonography, with a maximum deviation of 0.07 cm from tape measurements, demonstrating that the sonographic measurements are equivalent to what can be expected post mortem under slaughterhouse conditions.

### 2.3. Litter Characteristics

Litter characteristics (that is total and life born piglets) and individual piglet birth weights were recorded within 24 h postpartum (using a hanging scale; Dr. meter, Hong Kong, China).

### 2.4. Statistical Analysis

Data processing and initial calculations were performed using Microsoft Excel 2021 (Microsoft Corporation, Redmond, WA, USA). Statistical analyses were conducted with GraphPad Prism 9 software (GraphPad Software, Boston, MA, USA). To adjust for piglet size variations, which are known to occur in larger litters, an attempt was made to calculate the mean values of the paired measurements per sow or gilt. These mean values were subsequently used for all analyses to account for the lack of independence between fetuses from the same pregnancy. Normality of fetometric measurements was evaluated using the D’Agostino–Pearson, Anderson–Darling, and Shapiro–Wilk tests. If any of these three tests failed to confirm normal distribution for a given parameter on any examination day, the parameter was considered not normally distributed overall; consequently, a non-normal distribution was assumed for the entire dataset. Results were compared for genetic group, litter size, and parity. Possible differences between low- and medium- to high-prolificacy genotypes were assessed using the Mann–Whitney U test, and potential influences of litter size and parity on fetal size parameters were evaluated using Spearman’s rank correlation coefficient (rs), complemented by simple linear regression analysis. Differences were considered statistically significant at *p* < 0.05. Due to the limited availability of sows of the low-prolificacy genotype (German Saddleback), which is rare and only kept on few farms, no formal sample size or power analysis could be performed. The number of animals included reflects the maximum available sample from the farm under study.

## 3. Results

### 3.1. Litter Characteristics

Breeding performance differed significantly between the groups (*p* = 0.0027), with a mean total number of piglets born of 10.87 in the low-prolificacy group (GS) and 14.37 in the medium- to high-prolificacy group (GL/DuGL) (Table 1).

Mean birth weight did not differ between low-prolificacy group and medium- to high-prolificacy group (Mean: 1431.79 g; Min: 365 g; Max: 2475 g versus Mean: 1369.47; Min: 260 g; Max: 2375 g; *p* = 0.6928).

### 3.2. Results of Fetometric Parameters

One low-prolificacy sow (German Saddleback) experienced abortion on gestation day 62, reducing the number of pregnancies included in subsequent measurements from 70 to 69.

**Figure 2:**
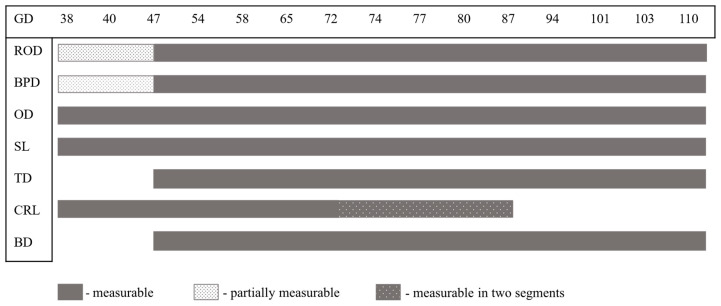
Feasibility of measuring fetometric parameters in pig fetuses during gestation. GD = gestation day; ROD = rosto-occipital distance; BPD = biparietal distance; OD = orbital distance; TD = thorax diameter; SL = sternum length; CRL = crown–rump length; BD = body diameter.

The feasibility of recording the seven fetometric parameters throughout gestation changed individually for each parameter, as the visibility of the fetuses due to, e.g., inappropriate fetal positioning limited some measurements (Figure 2).

During early gestation (gestation days 38 and 40), either ROD or BPD could typically be measured in a given fetus, due to the small fetal size and relatively large amniotic fluid volume, which restricted the ability to alter fetal positioning and thus confined measurements to the available imaging plane. TD and BD were generally measurable from day 47 onward, as complete visualization of the sternum and vertebral column, or the lumbar spine, was required. CRL could be recorded within a single sonographic image until approximately day 72 (penetration depth 18 cm); for larger fetuses exceeding the maximum size of the screen along the longitudinal axis, CRL was obtained in two segments until around day 77 (*n* = 81). From day 87 onward, CRL measurement was not feasible due to the enlarged fetal size.

Mean values of the two fetuses per sow and examination were calculated for all subsequent analyses. Absolute differences between the paired fetuses on gestation days 38 and 77 are presented in Table 2. These values reflect deviations in the measurements of fetometric parameters within the same sow and examination day. Days 38 and 77 correspond to the calculated boundaries of the first and second trimesters, respectively. No differences were observed between paired fetuses for ROD, BPD, and OD on day 38, where as the maximum difference was found for CRL on day 77, reaching 2.9 cm.

Fetometric measurements obtained throughout gestation are summarized in Table 3 including data for day 103, which represents approximately 90% of gestation. All parameters increased as gestation progressed. For instance, CRL representing the most practical and commonly used parameter for estimating gestational age ex vivo in slaughterhouses, increased from a median of 3.2 cm (range 1.9–4.2 cm) at day 38 to 16.3 cm (range 14.0–18.2 cm) at day 77. Measurements from the remaining 12 examination days are provided in the Supplementary Material.

**Figure 3:**
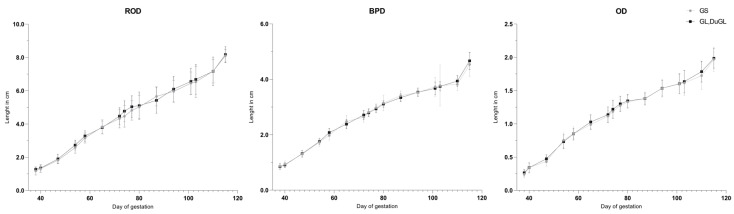
Gestational growth trajectories of the skull measurements. ROD = rosto-occipital distance; BPD = biparietal distance; OD = orbital distance; GS = German Saddleback; GL, DuGL = German Landrace, Duroc × German Landrace hybrids.

**Figure 4:**
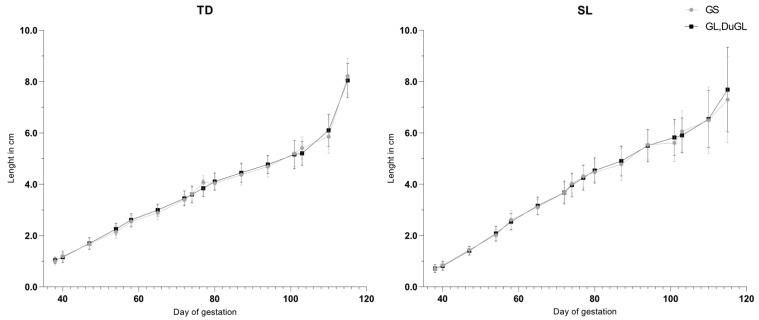
Gestational growth trajectories of the thoracic measurements.SL = sternum length; TD = thorax diameter; GS = German Saddleback; GL, DuGL = German Landrace, Duroc × German Landrace hybrids.

**Figure 5:**
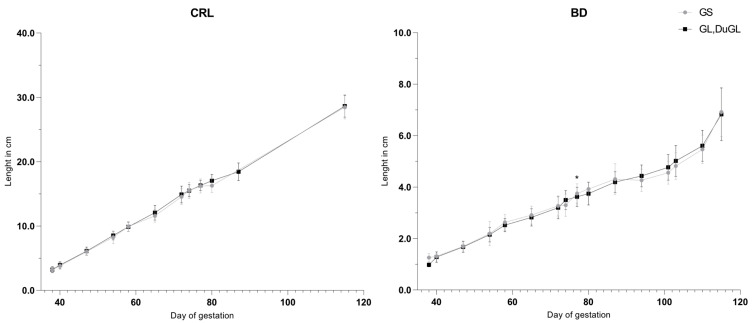
Gestational growth trajectories of the body measurements. CRL = crown–rump length; BD = body diameter; GS = German Saddleback; GL, DuGL = German Landrace, Duroc × German Landrace hybrids.

No significant differences in the recorded fetometric parameters were identified between the low-prolificacy (GS) and medium- to high-prolificacy (GL and DuGL) genotypes (Figure 3, Figure 4 and Figure 5). Overall, growth trajectories of the skull, thoracic, and body measurements were comparable across groups, with the sole exception of BD, which was significantly greater in the low-prolificacy genotype compared to the medium- to high-prolificacy genotype on day 77 of gestation (*p* = 0.0465).

### 3.3. Influencing Factors

Across all examination days, litter size exerted no or only minimal influence on the majority of fetometric parameters assessed. Specifically, no significant associations were detected for ROD (*p* = 0.3593; *n* = 65), BPD (*p* = 0.8313; *n* = 65), OD (*p* = 0.6603; *n* = 65), SL (*p* = 0.1076; *n* = 65), CRL (*p* = 0.3456; *n* = 57), or BD (*p* = 0.0934; *n* = 65). Only on day 77 of gestation, a correlation between litter size and TD was observed (*p* = 0.0331; rs = −0.2647; *n* = 65). Regarding parity (range 1–9), correlation analysis did not reveal any statistically significant impact on skull parameters (ROD: *p* = 0.0606; BPD: *p* = 0.9755; OD: *p* = 0.1867), thoracic measurements (TD: *p* = 0.3513; SL: *p* = 0.6505), or body parameters (CRL: *p* = 0.1082; BD: *p* = 0.3322).

## 4. Discussion

This is the first study to comprehensively assess swine fetal age via sonographic fetometry. Although fetometric data is established in other species, its direct transfer to pigs is limited due to marked differences in gestation length, fetal growth dynamics, litter size, and placentation. Consequently, species-specific reference data is essential. As a non-invasive method, it is of particular ethical relevance for age determination. Sonography, a well-established diagnostic tool in swine reproduction [41,42], allowed the collection of clearly defined and consistently applicable fetometric measurements, providing a solid basis for fetal assessment. Parameters with distinct anatomical landmarks enabled reproducible measurements. The study design incorporated short video sequences during sonography, permitting retrospective selection of the most accurate frames. Combined with clearly defined anatomical parameters, this approach enhances both the validity and reproducibility of the measurements.

The differences in breeding performance between the low- and medium- to high-prolificacy genotypes support their separation for subsequent analyses.

Among the seven parameters evaluated, OD and SL were measurable across the entire observation period (38–110 GD), although the relatively small range of OD (0.2–2.2 cm) may limit its practical utility. CRL was measurable up to approximately day 87 of gestation, with comparability between direct single-frame and segmental measurements, as originally described by Khand et al. [29] for caprine fetus. Beyond day 87, CRL could no longer be assessed sonographically, whereas the remaining parameters—BPD, ROD, TD, SL, BD, and OD—remained measurable up to day 110, supporting gestational age estimation throughout most of gestation. During the transition from the first to the second trimester, CRL, OD, and SL were particularly informative, whereas in the final trimester, CRL became impractical, and the remaining six parameters allowed reliable age determination.

Despite the higher total number of piglets born in the medium- to high-prolificacy genotype compared to the low-prolificacy genotype, no significant differences in fetal growth parameters could be demonstrated, based on a sample size of 70 pregnancies, with two fetuses per pregnancy. Recorded birth weights did not differ significantly between low-prolificacy and medium- to high-prolificacy sows and showed considerable variation, reflecting substantial intra-litter variability. Very low birth weights (<400 g), may be attributable to intrauterine growth retardation (IUGR), which occurs more frequently in large litters and can reduce individual fetal birth weights [43,44]. A plausible explanation for the absence of significant differences in birth weight between genotypes is an inherent limit of uterine and placental capacity. König et al. [45] demonstrated that, despite larger litter sizes in high-prolificacy sows, placental efficiency was increased such that individual fetal growth was largely maintained. This adaptive capacity buffers the effects of increased litter size, resulting in comparable birth weights and, consequently, explains why fetometric parameters measured in vivo also did not differ significantly across genotypes. Limited uterine and placental capacity imposes constraints on fetal development, and intrauterine competition for nutrients and oxygen can generate a broad distribution of birth weights while still allowing generally uniform growth of fetal organs and body dimensions [20,44,45]. The temporal dynamics of fetal growth further contribute to this effect. Body growth progresses relatively slowly during early and mid-gestation and accelerates in the final third of pregnancy, when fetal weight typically rises exponentially [46]. Fetal growth is influenced not only by maternal genotype and litter size but also by molecular regulatory mechanisms, including microRNAs and their target genes, which act independently of genotype and play a central role in prenatal skeletal muscle development and overall fetal growth [47]. These genotype-independent molecular processes likely contribute to the observed consistency of fetometric parameters across the investigated sow lines, despite the wide variability in birth weights.

The present study included sow genotypes commonly used in German production systems—German Landrace, Duroc—but these represent only part of the genetic spectrum. Other maternal lines with medium- to high-prolificacy potential, such as Norwegian Landrace or Large White, were not included. Nevertheless, as no significant differences in fetometric parameters were observed among the investigated genotypes, this consistency likely reflects not only the comparable prolificacy of the lines but also underlying molecular regulatory mechanisms, such as microRNAs influencing prenatal growth, which act independently of genotype. Therefore, the measurements provide a reliable basis for fetal age estimation across a broad range of commercial sow populations. Whether the presented data are applicable to hyperprolific breeds, such as Danish Landrace, remains to be investigated. Future studies including such genotypes are necessary to validate the reference values under conditions of extreme litter sizes and enhanced reproductive performance.

Compared with existing German reference data, this study reports lower CRLs: 3.2 cm (range 1.9–4.2 cm) at day 40 and 16.3 cm (range 14.0–18.2 cm) at day 80, versus established values of 5 cm and 21 cm [15,16], respectively. These discrepancies are likely due to shifts in breeding goals over recent decades: whereas older selection programs emphasized fetal size and mass, modern commercial breeding increasingly prioritizes litter size, which may result in smaller fetal dimensions [48]. Scandinavian data reported by Gjesdal (1972) describe cranial length, which corresponds to the rosto- occipital distance assessed in the present study [49]. This was defined as the distance between the nasal border of the incisive bone and the occipital crest, measured in sagittally sectioned heads using radiographic imaging. In pig fetuses between days 76 and 80 of gestation, values ranged from 6.1 to 6.7 cm, clearly exceeding the median rostro-occipital distance of 4.9 cm observed in the present investigation. These differences further support the notion that fetal dimensions have decreased over time, likely reflecting ongoing changes in breeding strategies. No comparative reference values exist for TD, SL, BD, or OD. The measurements obtained in this study thus provide a more contemporary and practically relevant basis for gestational age estimation in swine fetuses.

Nevertheless, several limitations should be considered. The assessment of only two fetuses per litter inherently limits the capture of intra-litter variability. As measurements of the two fetuses occasionally differed, mean values were calculated to provide representative estimates of fetal size, while slightly reducing precision compared with individual measurements. Early gestation (days 38–40) was constrained by incomplete ossification, limiting visualization of structures such as the vertebral column, while late gestation restricted assessment of crown–rump length due to the fetus exceeding the ultrasound field. Fetal position variability and the inability to mark or consistently re-identify individual fetuses may have introduced additional measurement uncertainty, as different fetuses were likely examined on each examination day. Although it has been shown that individual fetuses can be reliably identified in vivo during the last ten days of gestation [50], this was not feasible in the present study, as measurements were performed across the entire gestation period. The study was conducted on a single farm with a small sample size (*n* = 70 pregnancies) and a limited genetic background, excluding highly prolific breeds, which may limit generalizability. Slight variations in ovulation timing following artificial insemination may have led to differences of around one day in actual gestational age, representing a minor, inherent consideration when applying standardized age estimates.

The ethical relevance of this diagnostic approach is considerable. Sonographic fetometry offers a practical, non-lethal alternative to the invasive and ethically problematic practice of culling potentially pregnant sows and fetuses for gestational age determination, aligning with Germany’s constitutional goal of animal welfare. From a legal perspective, the study addresses a knowledge gap in the enforcement of Council Regulation (EC) No. 1/2005 [7] and the German Animal Products Trade Prohibition Act [8] with regard to sows.

The fetometric measurements obtained here provide herd veterinarians with a scientifically robust and practicable tool for supporting management decisions concerning pregnant sows. Moreover, this method, which relies on fetometric parameters well suited for measurement on the slaughter line, provides a sound basis for veterinary authorities to assess the legality of animal transport and subsequent slaughter and could be implemented in abattoir settings.

## 5. Conclusions

The fetometric parameters identified in this study enable a scientifically sound estimation of fetal age in pigs and thus offer an objective tool to support animal welfare enforcement in suspected violations of regulations governing the transport and slaughter of pregnant animals. The findings suggest that a CRL of ≥3.2 cm is indicative of the second trimester, whereas a CRL of ≥16.3 cm corresponds to the third trimester of gestation. In cases where gestation is suspected to exceed 90% of the total gestation period, additional fetometric parameters, including ROD, BPD, TD, SL, BD, should be considered for age estimation. Further research in additional breeds, particularly hyperprolific genotypes, may be warranted.

## Figures and Tables

**Table 1:** Total number of piglets born and mean birth weights in low and medium- to high-prolificacy genotypes.

Litters (*n*)	SD Birth Weight (g)	Mean Birth Weight (g)	Max. Total Born	Min. Total Born	SD Total Born	Mean Total Born	Genotype
19	385.56	1431.79	18	4	3.54	10.87 ^a^	Low-prolificacy (GS)
50	384.1	1369.47	26	7	5.73	14.37 ^b^	Medium- to high-prolificacy (GL, DuGL)

SD = standard deviation; GS = German Saddleback; GL, DuGL = German Landrace, Duroc × German Landrace hybrids. ^a b^ significant difference (*p* = 0.0027).

**Table 2:** Absolute differences between paired fetuses measured within the same sow on gestation days 38 and 77.

*n*	Max. Diff. (cm)	Min. Diff. (cm)	Median Diff. (cm)	GD	Parameter
11	0.39	0	0.14	38	ROD
41	0.39	0	0.1		BPD
69	0.14	0	0.05		OD
3	0.27	0.11	0.14		TD
67	0.66	0	0.08		SL
66	1.19	0.01	0.29		CRL
3	0.2	0.1	0.15		BD
69	1.09	0.01	0.26	77	ROD
69	0.68	0	0.16		BPD
69	0.55	0.01	0.12		OD
69	1.06	0	0.26		TD
69	1.34	0.01	0.29		SL
61	2.9	0.03	0.76		CRL
69	1.34	0.01	0.28		BD

GD = gestation day; ROD = rosto-occipital distance; BPD = biparietal distance; OD = orbital distance; TD = thorax diameter; SL = sternum length; CRL = crown–rump length; BD = body diameter; Median Diff. = median absolute difference (cm); Min./Max. Diff.= minimum/maximum absolute difference (cm); *n* = number of paired measurements. Of the 70 pregnancies, one sow experienced an abortion on day 62, reducing the total number to 69 measurements.

**Table 3:** Results of measurements of fetometric parameters at days 38, 77, and 103 of gestation.

*n*	Max. (cm)	Min. (cm)	Median (cm)	GD	Parameter
44	1.8	1	1.3	38	ROD
64	1.1	0.6	0.9		BPD
70	0.4	0.1	0.3		OD
11	1.2	0.7	1		TD
69	1.5	0.5	0.7		SL
70	4.2	1.9	3.2		CRL
7	1.3	0.9	1.1		BD
69	6	3.3	4.9	77	ROD
69	3.2	2.6	2.9		BPD
69	1.5	1	1.3		OD
69	4.7	3	3.9		TD
69	5.7	3.3	4.3		SL
61	18.2	14	16.3		CRL
69	4.7	2.9	3.7		BD
69	8.1	4.8	6.6	103	ROD
69	4.5	3.3	3.9		BPD
69	2	1.2	1.6		OD
69	6.3	4.3	5.3		TD
69	8.2	4.6	6		SL
n.m.	n.m.	n.m.	n.m.		CRL
69	6.3	3.5	4.9		BD

GD = gestation day; ROD = rosto-occipital distance; BPD = biparietal distance; OD = orbital distance; SL = sternum length; TD = thorax diameter; CRL = crown–rump length; BD = body diameter; Min./Max. = minimum/maximum (cm); n = number of paired measurements; n.m. = not measurable. Of the 70 pregnancies, one sow experienced an abortion on day 62, reducing the total number to 69 measurements.

## Data Availability

All data are available from the corresponding author upon reasonable request.

## Supplementary Materials

The following supporting information can be downloaded at: https://www.mdpi.com/article/10.3390/ani16020349/s1, Table S1: Fetometric parameters measured at days 40, 47, 54, 58, 65, 72, 74, 80, 87, 94, 101 and 110 of gestation.
