# Supplementary material for: Sonographic Assessment of Fetometric Parameters in Pigs of Different Prolific Genotypes for Gestational Age Estimation
Source: Animals (Basel). 2026 Jan 22;16(2):349. doi: 10.3390/ani16020349 (PMC12837421; doi:10.3390/ani16020349)
Supplement: Supplementary file 1 [file animals-16-00349-s001.zip › animals-4075644-supplementary.pdf]

Supplementary Material

Table S1 Fetometric parameters measured at days 40, 47, 54, 58, 65, 72, 74, 80, 87, 94, 101 and 110 of gestation.

Parameter	GD	Median (cm)	Min. (cm)	Max. (cm)	n
ROD	40	1.3	0.7	1.7	46
BPD		0.9	0.7	1.2	65
OD		0.3	0.2	0.5	69
TD		1.1	0.7	1.5	27
SL		0.9	0.5	1.4	70
CRL		3.8	2.2	5.0	70
BD		1.3	1.0	1.7	18
ROD	47	1.9	1.4	2.6	69
BPD		1.3	1.0	1.6	70
OD		0.5	0.3	0.6	70
TD		1.6	1.0	2.1	70
SL		1.4	0.8	1.7	70
CRL		6.0	4.4	7.6	70
BD		1.7	1.2	2.2	70
ROD	54	2.7	1.9	3.3	70
BPD		1.6	1.4	2.0	70
OD		0.8	0.6	1.1	70
TD		2.2	1.6	2.7	70
SL		2.1	1.6	2.9	70
CRL		8.4	6.0	9.7	70
BD		2.2	1.5	3.8	70
ROD	58	3.2	2.2	4.0	70
BPD		2.1	1.7	2.4	70
OD		0.9	0.6	1.1	70
TD		2.6	1.9	3.0	70
SL		2.5	1.8	3.4	70
CRL		9.8	7.8	11.4	70
BD		2.6	2.0	3.2	70
ROD	65	3.8	2.4	4.5	69
BPD		2.4	2.1	3.1	69

OD		1.0	0.8	1.4	69
TD		3.0	2.3	3.5	69
SL		3.1	2.2	4.1	69
CRL		12.0	10.0	14.0	69
BD		2.9	2.4	3.9	69
ROD		4.3	2.9	5.2	69
BPD		2.7	2.2	3.0	69
OD		1.1	0.8	1.4	69
TD	72	3.4	2.8	4.0	69
SL		3.7	2.6	4.5	69
CRL		14.7	11.1	16.8	62
BD		3.3	2.4	4.3	69
ROD		4.6	2.8	5.7	69
BPD		2.8	2.4	3.0	69
OD		1.2	0.8	1.5	69
TD	74	3.6	2.8	4.3	69
SL		3.9	3.1	4.9	69
CRL		15.2	12.8	16.8	61
BD		3.5	2.8	4.4	69
ROD		5.2	3.7	6.9	69
BPD		3.1	2.8	4.2	69
OD		1.3	1.1	1.6	69
TD	80	4.0	3.3	4.7	69
SL		4.5	3.5	5.7	69
CRL		16.7	14.0	18.2	36
BD		3.9	3.1	4.7	69
ROD		5.6	4.1	7.1	69
BPD		3.4	3.0	3.8	69
OD		1.4	1.2	1.6	69
TD	87	4.5	3.6	5.4	69
SL		4.0	4.1	6.7	69
CRL		18.7	17.2	20.4	6
BD		4.2	3.3	6.2	69

ROD		6.0	4.6	7.7	69
BPD		3.5	3.1	3.9	69
OD		1.5	1.2	1.8	69
TD	94	4.8	3.8	5.6	69
SL		5.5	4.4	7.1	69
CRL		n.m.	n.m.	n.m.	n.m.
BD		4.5	3.6	5.6	69
ROD		6.4	4.3	7.9	69
BPD		3.7	3.2	5.0	69
OD		1.6	1.1	1.8	69
TD	101	5.2	3.6	6.4	69
SL		5.9	4.3	7.8	69
CRL		n.m.	n.m.	n.m.	n.m.
BD		4.8	3.8	5.9	69
ROD		7.1	4.6	8.8	68
BPD		3.9	3.4	4.5	69
OD		1.8	1.2	2.1	69
TD	110	6.0	4.8	7.2	69
SL		6.9	5.4	10.9	69
CRL		n.m.	n.m.	n.m.	n.m.
BD		5.6	4.2	7.1	69

GD = gestation day; ROD = rostro-occipital distance; BPD = biparietal distance; OD = orbital distance; SL = sternum length; TD = thorax diameter; CRL = crown–rump length; BD = body diameter; Min./Max. = minimum/maximum (cm); n = number of paired measurements; n.m. = not measurable. Of the 70 pregnancies, one sow experienced an abortion on day 62, reducing the total number to 69 measurements. CRL could be recorded within a single sonographic image until approximately day 72 and thereafter using two segments up to approximately day 77.
